# Molecular Network Analysis Discloses the Limited Contribution to HIV Transmission for Patients with Late HIV Diagnosis in Northeast China

**DOI:** 10.1007/s10508-022-02492-4

**Published:** 2022-12-20

**Authors:** Bin Zhao, Wei Song, Mingming Kang, Xue Dong, Xin Li, Lu Wang, Jianmin Liu, Wen Tian, Haibo Ding, Zhenxing Chu, Lin Wang, Yu Qiu, Xiaoxu Han, Hong Shang

**Affiliations:** 1 NHC Key Laboratory of AIDS Immunology (China Medical University), National Clinical Research Center for Laboratory Medicine, The First Hospital of China Medical University, No 155, Nanjing North Street, Heping District, Shenyang, 110001 Liaoning Province China; 2 Laboratory Medicine Innovation Unit, Chinese Academy of Medical Sciences, Shenyang, China; 3 Key Laboratory of AIDS Immunology of Liaoning Province, Shenyang, China; 4 Collaborative Innovation Center for Diagnosis and Treatment of Infectious Diseases, Hangzhou, China; 5 Department of Food Safety and Nutrition, Shenyang Center for Health Service and Administrative Law Enforcement (Shenyang Center for Disease Control and Prevention), Shenyang, China

**Keywords:** HIV, Late diagnosis, Subtype, Molecular network, China

## Abstract

In the “treat all” era, the high rate of late HIV diagnosis (LHD) worldwide remains an impediment to ending the HIV epidemic. In this study, we analyzed LHD in newly diagnosed people living with HIV (PLWH) and its impact on HIV transmission in Northeast China. Sociodemographic information, baseline clinical data, and plasma samples obtained from all newly diagnosed PLWH in Shenyang, the largest city in Northeast China, between 2016 and 2019 were evaluated. Multivariate logistic regression analysis was performed to identify risk factors associated with LHD. A molecular network based on the HIV *pol* gene was constructed to assess the risk of HIV transmission with LHD. A total of 2882 PLWH, including 882 (30.6%) patients with LHD and 1390 (48.2%) patients with non-LHD, were enrolled. The risk factors for LHD were older age (≥ 30 years: *p* < .01) and diagnosis in the general population through physical examination (*p* < .0001). Moreover, the molecular network analysis revealed that the clustering rate (*p* < .0001), the fraction of individuals with ≥ 4 links (*p* = .0847), and the fraction of individuals linked to recent HIV infection (*p* < .0001) for LHD were significantly or marginally significantly lower than those recorded for non-LHD. Our study indicates the major risk factors associated with LHD in Shenyang and their limited contribution to HIV transmission, revealing that the peak of HIV transmission of LHD at diagnosis may have been missed. Early detection, diagnosis, and timely intervention for LHD may prevent HIV transmission.

## Introduction

The long incubation period from HIV infection to AIDS and the lack of specific symptoms of early HIV infection delay diagnosis in some patients. In 2013, the concept of late HIV diagnosis (LHD) was developed based on the CD4 + T cell count (< 350 cells/µl) within 3 months from the date of diagnosis (Kozak et al., 2013). The estimated median time from HIV infection to the aforementioned threshold of CD4 + T cells is 4 years (Lodi et al., 2011). LHD allows the disease to progress undisturbed to AIDS, thereby increasing the mortality rate and the transmission of HIV. In the “treat all” era, LHD is a major obstacle to ending the HIV epidemic. Unfortunately, LDH is very common globally. In 2020, almost half of all new infections in Europe were LHD (European Centre for Disease Prevention and Control/WHO Regional Office for Europe, 2021). In the USA, the percentage of LHD ranges from 39 to 53% (Nduaguba et al., 2021; Wilton et al., 2019). In China, a recent study showed that the prevalence of LHD reached 43% (Sun et al., 2021).

It is well established that LHD can lead to poorer individual health outcomes and increase health care costs (Guaraldi et al., 2017). Research has also provided insight into the characteristics of LHD. By understanding the features of HIV, testing interventions can directly target them to reduce the prevalence of LHD. The study based on the EuResist database between 1981 and 2019 showed that the risk factors for LHD included advanced age (> 56 years), heterosexual contact, an African origin, and a log viral load (VL) > 4.1 (Miranda et al., 2021). Another recent study conducted in the USA reported that older individuals and Hispanics were at increased risk for LHD (Nduaguba et al., 2021). A recently published systematic review showed that the factors associated with LHD in China were patient age ≥ 50 years, marriage, and diagnosis in medical institutions (Sun et al., 2021).

The lack of antiretroviral therapy and the presence of an uncontrolled VL in patients with LHD increases the risk of HIV transmission to others. However, there is limited knowledge regarding the negative impact of LHD on public health. Molecular network analysis based on the *pol* gene sequences can clarify the mode of HIV transmission and guide targeted intervention (Wertheim et al., 2014; Zhao et al., 2021a, 2021b). In addition, it can be used to evaluate the risk of HIV transmission and the contribution of individuals to this transmission within the network (Little et al., 2014). Thus far, only one Danish study, using phylogenetic analysis, has revealed that the risk of LHD was lower for active clusters compared with non-LHD (van Wijhe et al., 2021). To date, molecular network technology has rarely been used to explore the impact of LHD on HIV onward transmission.

Shenyang, the largest city in Northeast China, has a moderate HIV prevalence (> 10,000 people living with HIV [PLWH]) (Wu et al., 2019). Men who have sex with men (MSM) accounted for 80.8% of new HIV infections in Shenyang (Jinping et al., 2018). Some larger, urban cities (e.g., Shanghai, Beijing, Tianjin, and Shenzhen) are also characterized by moderate or high HIV endemicity, mainly attributed to MSM (Jinping et al., 2018). This is inconsistent with the national HIV infection situation, which is dominated by heterosexual transmission. Previous studies have also found a close relationship between main epidemic strains (CRF01_AE and CRF07_BC) associated with infection among MSM in these cities (Han et al., 2013). Therefore, it is necessary to investigate the situation of LHD in Shenyang. The results can be extrapolated to other cities with similar epidemic characteristics. The objective of this study was to investigate the risk factors of LHD and identify the impact of LHD on HIV transmission in Shenyang from 2016 to 2019, aiming to guide targeted HIV detection and appropriate intervention.

## Method

### Participants

This study involved an observational cohort, which included all newly diagnosed patients with HIV infection in Shenyang from 2016 to 2019 (Zhao et al., 2021a, 2021b). Baseline demographic information, baseline VL, CD4 + T cell counts, and available cryopreserved plasma samples collected at diagnosis were obtained from the Red Ribbon Outpatient of the First Affiliated Hospital of China Medical University (Shenyang, China) and Shenyang Center for Disease Control and Prevention (Shenyang, China).

The patients included in this study were: (1) newly diagnosed with HIV between 2016 and 2019, and (2) antiretroviral therapy-naïve (self-reported) before the diagnosis of HIV. Individuals without successful *pol* gene sequencing were excluded.

Most newly diagnosed individuals were classified as recent HIV infection (RHI) or chronic HIV infection according to the result of the HIV-1 Limiting Antigen Avidity test (Maxim Biomedical, 2013). The details of the experiment have been previously described in the published literature (Zhao et al., 2021a, 2021b). In this study, patients identified with chronic HIV infection and whose baseline CD4 + T cell count was < 350 cells/µl (Kozak et al., 2013) were classified as LHD; patients identified with RHI or with a baseline CD4 + T cell count ≥ 350 cells/µl were classified as non-LHD (NLHD).

### Measures

#### Sequence Analysis

The HIV *pol* sequences (HXB2: 2253–3318) were obtained for the analysis of HIV drug resistance using an in-house method (Zhao et al., 2011). The subtypes of HIV-1 subtype were analyzed and determined based on the neighbor-joining tree containing reference sequences under the Kimura two-parameter model with 1000 bootstrap replicates using MEGA v7.0.14 (Kumar et al., 2016). The reference sequences including major subtypes and some common recombinants in China were downloaded from the Los Alamos database (http://www.hiv.lanl.gov/). Potential recombinants of the sequences were identified using the Recombination Identification Program (RIP) v3.0 (Siepel et al., 1995). For a more detailed analysis, please refer to a previous publication by Zhao et al. (2021a, 2021b).

#### HIV Transmission Risk Analysis

Briefly, a previous study confirmed the optimal genetic distance threshold of major circulating HIV strains (CRF01_AE [69.9%, 2019/2887]; CRF07_BC [18.2%, 526/2887]; and B [4.6%, 132/2887]) through sensitivity analysis (Zhao et al., 2021a, 2021b). Molecular networks of three major circulating HIV stains in Shenyang have been constructed (Zhao et al., 2021a, 2021b) based on pairwise nucleotide genetic distance using the obtained optimal genetic distance threshold and HIV-TRACE (TRAnsmission Cluster Engine) (Kosakovsky Pond et al., 2018). Cytoscape v3.7.2 (Shannon et al., 2003) was used to visualize the networks.

Three indicators were used to assess the risk of HIV transmission. Firstly, the clustering rate (individuals in the network/all individuals included in the molecular network analysis) was the most direct indicator for assessing the risk of HIV transmission in the population. Secondly, the number of links in the network was used to assess an individual’s risk of HIV transmission. The third quartile of the number of links for all individuals with > 1 link is usually used as the threshold for determining the risk (Little et al., 2014). The proportion of individuals with a high number of links in the population (i.e., individuals with a certain number of links/all individuals included in the molecular network analysis) can also reflect the risk of HIV transmission for a group. Thirdly, in practice, contributions to RHI infection (links to RHI in networks) were also used to assess an individual’s risk of HIV transmission; this may be a more accurate and specific indicator. The proportion of individuals contributing to RHI (individuals contributing to RHI/all individuals included in the molecular network analysis) was compared to reflect the risk of HIV transmission between LHD and NLHD. Of note, newly diagnosed RHI in a certain year (e.g., 2017) can only be infected by individuals diagnosed with HIV during the same or the previous year (i.e., 2016–2017).

### Statistical Analyses

The chi-squared test was used to compare the categorical variables between the LHD and NLHD groups. An independent-sample *t* test was performed to compare continuous variables (VL and CD4 + T cell count) between the LHD and NLHD groups. Multivariate logistic regression analysis was performed to identify risk factors associated with LHD. Demographic factors including gender, age, race, marital status, education level, household register location, and behavioral factors including infection route and sample sources were controlled as confounding factors. In the univariate logistic model, one independent variable with *p* < .2 was entered into the multivariable model each time. *P* values < .05 and < 0.10 denoted statistically significant and marginally significant differences, respectively. All statistical analyses were performed using the SPSS version 25.0 software (IBM Corp., Armonk, NY, USA).

## Results

### Study Population

A total of 2882 (88.1%, 2882/3272) patients who met the inclusion criteria were included in this study. Of those, 882 and 1390 patients were classified as LHD and NLHD, respectively. It was not possible to allocate the remaining 610 patients due to the lack of HIV-1 LAg-Avidity testing results and baseline CD4 + T cell counts. LHD accounted for 38.8% (882/2272) of newly diagnosed PLWH, and there was no significant difference in the annual proportion of LHD for the 4 years examined in this study (2016–2019) (Table 1).

**Table 1:** Sociodemographic and clinical characteristics of the included participants in Shenyang during 2016–2019

Characteristics	Total	Late HIV diagnosis	Non-late HIV diagnosis	*p* value
*Demographic information*				
Total	2272 (100.0)	882 (38.8)	1390 (61.2)	
Gender				
Male	2132 (93.8)	820 (93.0)	1312 (94.4)	.171
Female	140 (6.2)	62 (7.0)	78 (5.6)	
Age at enrollment (years)				
< 30	922 (40.6)	276 (31.3)	646 (46.5)	< .0001
30–39	661 (29.1)	283 (32.1)	378 (27.2)	
≥ 40	687 (30.3)	322 (36.5)	365 (26.3)	
Not available	2 (0.1)	1 (0.1)	1 (0.1)	
Race/ethnicity				
Han	1957 (86.1)	774 (87.8)	1183 (85.1)	.065
Others	314 (13.8)	107 (12.1)	207 (14.9)	
Not available	1 (0.0)	1 (0.1)	0 (0.0)	
Marital status				
Unmarried	1454 (64.0)	499 (56.6)	955 (68.7)	< .0001
Married/divorced/widower	811 (35.7)	378 (42.9)	433 (31.2)	
Not available	7 (0.3)	5 (0.6)	2 (0.1)	
Education level				
Junior high school and below	634 (27.9)	262 (29.7)	372 (26.8)	.114
Senior high school and above	1628 (71.7)	614 (69.6)	1014 (72.9)	
Not available	10 (0.4)	6 (0.7)	4 (0.3)	
Infection route				
Men have sex with men	1890 (83.2)	710 (80.5)	1180 (84.9)	.011
Heterosexual transmission	308 (13.6)	142 (16.1)	166 (11.9)	
Injection drug users	27 (1.2)	8 (0.9)	19 (1.4)	
Not available	47 (2.1)	22 (2.5)	25 (1.8)	
Household register location				
In Shenyang	1163 (51.2)	460 (52.2)	703 (50.6)	.217
In other cities in Liaoning Province	670 (29.5)	241 (27.3)	429 (30.9)	
In other provinces	330 (14.5)	118 (13.4)	212 (15.3)	
Not available	109 (4.8)	63 (7.1)	46 (3.3)	
Sample sources				
High-risk population for HIV screening^a^	1578 (69.5)	550 (62.4)	1028 (74.0)	< .0001
General population for physical examination^b^	615 (27.1)	311 (35.3)	304 (21.9)	
Blood donor for HIV detection	69 (3.0)	16 (1.8)	53 (3.8)	
Not Available	10 (0.4)	5 (0.6)	5 (0.4)	
Period of diagnosis				
2016	520 (22.9)	211 (23.9)	309 (22.2)	.595
2017	614 (27.0)	234 (26.5)	380 (27.3)	
2018	690 (30.4)	273 (31.0)	417 (30.0)	
2019	448 (19.7)	164 (18.6)	284 (20.4)	
*Clinical information*				
Subtypes				
CRF01_AE	1601 (70.5)	637 (72.2)	964 (69.4)	< .0001
CRF07_BC	427 (18.8)	124 (14.1)	303 (21.8)	
B	159 (7.0)	87 (9.9)	72 (5.2)	
Others	85 (3.7)	34 (3.9)	51 (3.7)	
Baseline viral load (log10 copies/mL)	4.7 ± 0.7 (*n* = 1573)	4.8 ± 0.7 (*n* = 722)	4.6 ± 0.7 (*n* = 851)	< .0001
Baseline CD4 + T cells (cells/μL)	305 ± 208 (*n* = 2018)	172 ± 105 (*n* = 882)	408 ± 210 (*n* = 1136)	< .0001
*Molecular network information*				
(CRF01_AE, CRF07_BC, and B)				
Total	2677 (100.0)	813 (30.4)	1312 (49.0)	
Clustering				
In the cluster	1158 (43.3)	299 (36.8)	617 (47.0)	< .0001
Not in the cluster	1519 (56.7)	514 (63.2)	695 (53.0)	
Links				
Links ≥ 4	170 (6.4)	45 (5.5)	98 (7.5)	.0847
Links < 4	2507 (93.6)	768 (94.5)	1214 (92.5)	
Contribution to recent HIV infection				
Linked to recent HIV infection	382 (14.3)	88 (10.8)	238 (18.1)	< .0001
Not linked to recent HIV infection	2295 (85.7)	725 (89.2)	1074 (81.9)	

^a^Sentinel monitoring, voluntary testing and consulting (VCT), sexually transmitted disease clinic, and high-risk population survey were included

^b^Routine physical examination, premarital examination, preoperative examination, and physical examination for enlistment, etc., were included

Among the 2272 patients, 93.8% (2132/2272) were male, and the median age was 32 years (interquartile range: 26–45 years). Furthermore, most patients were MSM (83.2%, 1890/2272), and 64.0% (1454/2272) were unmarried. Of note, 69.5% (1578/2272) individuals belonged to the high-risk population that underwent HIV screening; 27.1% (615/2272) individuals were identified from the general population through physical examination; and 3.0% (69/2272) patients were blood donors who were subjected to HIV testing. CRF01_AE accounted for the highest proportion of all detected subtypes (70.5%, 1601/2272). The average baseline VL was 4.7 ± 0.7 log_10_ copies/ml (*n* = 1573), and the average baseline CD4 + T cells was 305 ± 208 cells/μl (*n* = 2018) (Table 1).

Among the 882 patients with LHD, males accounted for 93.0% (820/882) of cases. The median age was 35 years (interquartile range: 28–47 years); 80.5% (710/882) were MSM and 56.6% (499/882) were unmarried; 62.4% (550/882) individuals belonged to the high-risk population that underwent HIV screening, and 35.3% (311/882) individuals were identified from the general population through physical examination. CRF01_AE accounted for 72.2% (637/882) of cases. The average baseline VL was 4.8 ± 0.7 log_10_ copies/ml (*n* = 722), and the average baseline CD4 + T cells was 172 ± 105 cells/μl (*n* = 882) (Table 1).

### Risk Factors Associated with Late HIV Diagnosis

Univariate logistic regression analysis showed that the patients with LHD tended to be older (age ≥ 30 years: *p* < .0001), belonged to other ethnic groups (non-Han) (*p* = .065), had been married at least once (*p* < .0001), were infected through heterosexual transmission (*p* = .004), and were identified from the general population through physical examination (*p* < .0001) (Table 2). All the aforementioned risk factors were included in the multivariate logistic regression analysis.

**Table 2:** Independent risk factors associated with late HIV diagnosis, Shenyang, 2016–2018 (*N* = 2272)

Demographic information	Univariate		Multivariate	
	Odds ratio (95% Confidence interval)	*p* value	Adjusted odds ratio (95% Confidence interval)	*p* value
*Gender*				
Male	Ref			
Female	1.27 (0.90–1.80)	.172		
*Age at enrollment (years)*				
< 30	Ref		Ref	
30–39	1.75 (1.42–2.16)	< .0001	1.58 (1.26–1.97)	< .0001
≥ 40	2.07 (1.68–2.54)	< .0001	1.60 (1.19–2.14)	.002
*Race/ethnicity*				
Han	Ref		Ref	
Other	0.79 (0.62–1.02)	.065	0.90 (0.70–1.17)	.436
*Marital status*				
Unmarried	Ref		Ref	
Married/divorced/widower	1.67 (1.40–1.99)	< .0001	1.11 (0.86–1.44)	.408
*Education level*				
Junior high school and below	Ref			
Senior high school and above	0.86 (0.71–1.04)	.114		
Infection route				
Men have sex with men	Ref		Ref	
Heterosexual transmission	1.42 (1.12–1.81)	.004	1.06 (0.81–1.38)	.671
Injection drug users	0.70 (0.31–1.61)	.400	0.52 (0.22–1.21)	.123
*Household register location*				
In Shenyang	Ref			
In other cities in Liaoning Province	0.86 (0.71–1.05)	.129		
In other provinces	0.85 (0.66–1.10)	.212		
*Sample sources*				
High-risk population for HIV screening^a^	Ref		Ref	
General population for physical examination^b^	1.91 (1.58–2.31)	< .0001	1.64 (1.34–2.00)	< .0001
Blood donor for HIV detection	0.56 (0.32–1.00)	.049	0.60 (0.34–1.06)	.080

^a^Sentinel monitoring, voluntary testing and consulting, sexually transmitted disease clinic, and high-risk population survey were included

^b^Routine physical examination, premarital examination, preoperative examination, and physical examination for enlistment, etc., were included

The multivariate logistic regression analysis showed that older age (30–39 years: adjusted odds ratio [aOR] = 1.578 [1.262–1.972], *p* < .0001; ≥ 40 years: aOR = 1.599 [1.194–2.140], *p* = .002) and diagnosis from the general population through physical examination (aOR = 1.635 [1.336–2.001], *p* < .0001) were risk factors associated with LHD (Table 2).

### HIV Transmission Risk Analysis

**Fig. 1:**
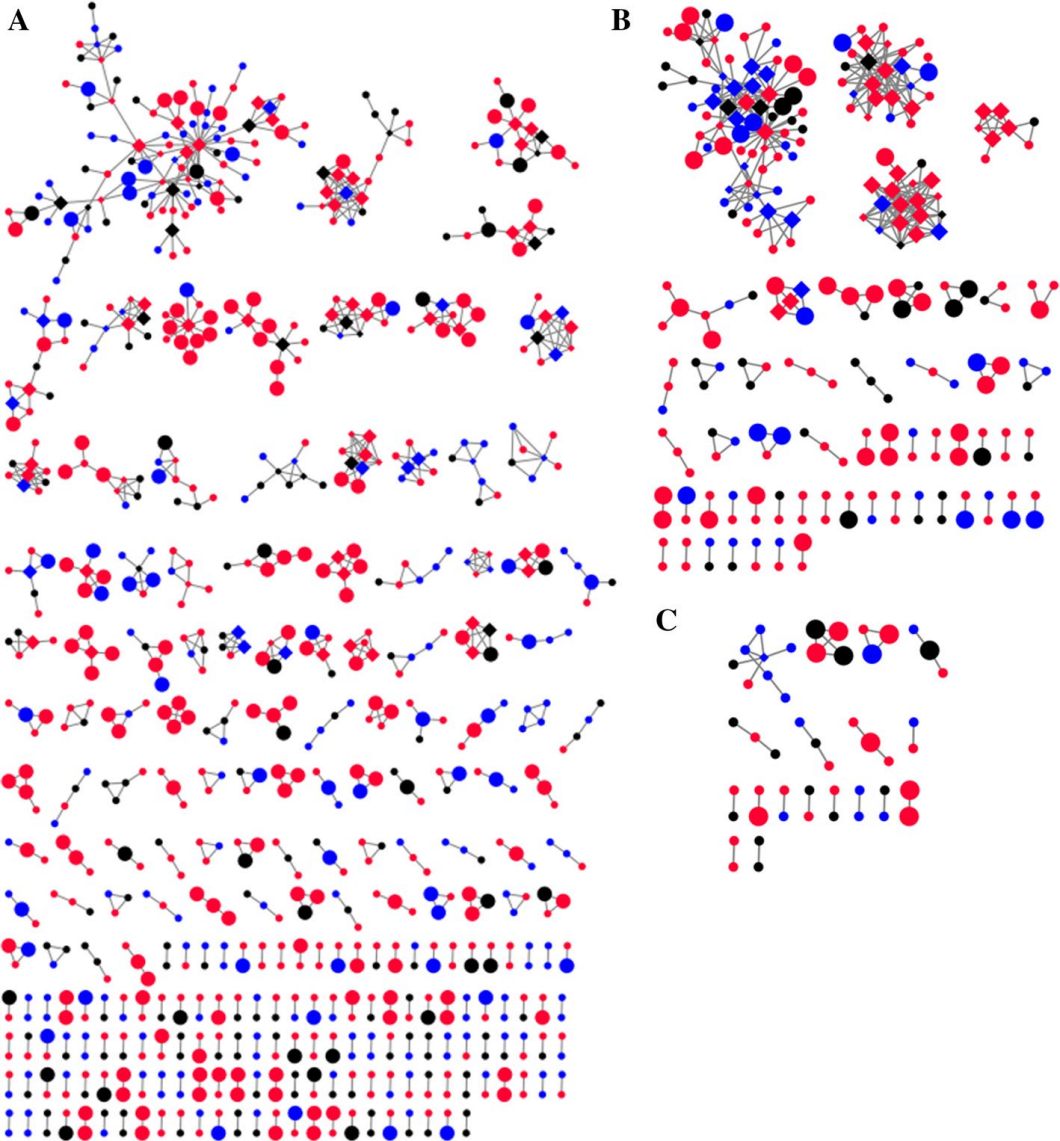
The molecular network diagram of major circulating HIV strains. **A** The molecular network of CRF01_AE. **B** the molecular network of CRF07_BC. **C** The molecular network of B. Blue nodes denote patients with late HIV diagnoses. Red nodes denote patients with non-late HIV diagnoses. Black nodes denote patients that could not be classified due to the lack of HIV-1 LAg-Avidity testing results and baseline CD4 + T cell counts. Squares denote patients with ≥ 4 links. Large nodes denote patients contributing to recent HIV infections (Color figure online)

As described earlier, 305 clusters (size range: 2–99) and 2606 links (range: 1–28) including 232 CRF01_AE clusters (857 sequences, 1760 links), 55 CRF07_BC clusters (252 sequences, 776 links), and 18 B clusters (49 sequences, 70 links) were inferred (Zhao et al., 2021a, 2021b) (Fig. 1).

Three indicators for assessing HIV transmission were analyzed. The clustering rate for patients with LHD was significantly lower than that of patients with NLHD (36.8% vs. 47.0%, respectively, *p* < .0001). In this study, ≥ 4 links was identified as the standard for high risk of HIV transmission. A total of 170 individuals with ≥ 4 links were identified in the network. The proportion of individuals with ≥ 4 links among patients with LHD was marginally significantly lower than that determined in patients with NLHD (5.5% vs. 7.5%, respectively, *p* = .0847). From 2017 to 2019, 298 RHI appeared in the molecular network, 88 patients with LHD led to 67 RHI, and 238 patients with NLHD led to 185 RHI. The contribution of individuals with LHD to RHI was significantly lower than that of patients with NLHD (10.8% vs. 18.1%, respectively, *p* < .0001) (Table 2).

## Discussion

In this study, we analyzed the prevalence of LHD in Shenyang from 2016 to 2019. We identified risk factors for LHD and assessed its contribution to HIV transmission through molecular networks, thus providing precise targets for HIV testing and interventions.

In this study, the overall prevalence of LHD in Shenyang was 38.8%, which is lower than the average level recorded in China (43.3%) (Sun et al., 2021) and markedly lower than those reported in Europe (50.0%) (Miranda et al., 2021) and the USA (39–53%) (Mao et al. 2018). This result may be related to the extensive HIV testing carried out in China, as well as recent research on HIV prevention and intervention performed in Shenyang in terms of the first real-world study of HIV pre-exposure prophylaxis in China (Wang et al., 2019), rapid clinical progression among acute HIV infection cases (Zhang et al., 2021a, 2021b), internet-based HIV self-testing among MSM (Zhang et al., 2021a, 2021b), and the relationship between assisted partner notification and uptake of HIV testing (Hu et al., 2021). These studies established a large HIV-negative high-risk MSM cohort in Shenyang and conducted regular HIV testing among them, thus possibly further reducing the incidence of LHD in this city.

In this study, the major risk factors for patients with LHD were older age (≥ 30 years) and diagnosis from the general population through physical examination. The results were similar to those of a recently published systematic review, but not entirely consistent. The review collected 39 Chinese publications on LHD from 2010 to 2020 and showed that the characteristics of LHD in China were patient age ≥ 50 years, marriage, heterosexual contact, and diagnosis in medical institutions (Sun et al., 2021). Although the present results indicated that age was associated with LHD, the patient age (≥ 30 years) was lower than that mentioned in the above study (i.e.,  ≥ 50 years). This may be related to the fact that the newly diagnosed PLWH in this study were mainly MSM, and there was a higher risk of HIV infection among young MSM (16–21 years) in China (Mao et al. 2018). Therefore, health care providers were less likely to consider an older MSM (≥ 30 years) infected with HIV. Moreover, older MSM (≥ 30 years) who have unsafe sex could still generally lack awareness concerning HIV testing (Trepka et al., 2014). Therefore, targeted publicity and educational activities should be carried out for different age groups.

The other risk factor identified in this study was the diagnosis from the general population through physical examination. In this study, patients diagnosed in the sexually transmitted disease clinic of medical institutions were included in the high-risk population for HIV screening. Similar to voluntary testing and counseling, most patients diagnosed in sexually transmitted disease clinics were also at high risk of HIV infection. Physical examination of the general population in this study included routine physical examination, premarital examination, preoperative examination, and physical examination for enlistment. This result suggests that numerous patients with LHD were undetected in the non-high-risk population, and the diagnosis of HIV infection among those individuals is challenging due to a lack of specific symptoms. Therefore, it is necessary to improve the identification of HIV-infected patients in routine medical services and provide passive HIV testing and consultation services for all individuals attending health facilities.

Thus far, few studies have evaluated the impact of LHD on public health. If individuals in the molecular network are well defined (e.g., availability of demographic information, antiretroviral therapy state, or the state of LHD, etc.), molecular network technology can evaluate the contribution of individuals to HIV transmission using network parameters (Little et al., 2014). Therefore, another important aspect of this study was the evaluation of the contribution of patients with LHD to HIV transmission using molecular network technology. We found that the contribution of patients with LHD to HIV transmission was significantly lower than that of patients with NLHD. This finding was similar to that reported in a recent Danish study, in which the risk for LHD was lower for active clusters compared to non-LHD (van Wijhe et al., 2021). Previous studies have shown that undiagnosed infected individuals, particularly those in the acute stage of HIV, could transmit the virus (Narasimhan & Kapila, 2019). It is easy to understand that, for patients with LHD, the peak of HIV transmission may have been missed at the time of HIV diagnosis. Based on this result, we can conclude that prompt diagnosis and intervention in patients with LHD may reduce the transmission of HIV.

Owing to its accuracy, the HIV-1 LAg-Avidity test was also included in the study as the basis for determining the LHD status of PLWH. However, this testing yielded some interesting results; for example, the CD4 + T cell count of some RHIs determined by the HIV-1 LAg-Avidity test was < 350 cells/µl. This result could be related to the high proportion of MSM infected with the CRF01_AE strain. This is because the disease may progress faster in patients infected with CRF01_AE than those with non-CRF01_AE (Liu et al., 2014). In addition, it may be related to the fact that the proportion of co-receptor CXCR4 of CD4 + T cells in AIDS patients infected with CRF01_AE is significantly higher than those observed in patients infected with other subtypes (Liu et al., 2014). Moreover, MSM with AIDS can experience a transformation of the co-receptor from C–C motif chemokine receptor 5 (CCR5) to C-X-C motif chemokine receptor 4 (CXCR4) in the early stage of HIV infection (Cui et al., 2019).

This study had some limitations. Firstly, the prevalence of LHD may be biased because it was not possible to classify 610 patients due to the lack of HIV-1 LAg-Avidity testing results and baseline CD4 + T cell counts. Secondly, in this study, LHD does not indicate late presentation for medical care against HIV, which reflects the disease stage only at the date of positive HIV diagnosis. Thirdly, as in all other similar molecular network studies, the inferred transmission link in the molecular transmission network does not represent HIV transmission relationships in the real world.

### Conclusions

Patients with LHD in Shenyang were characterized by older age (≥ 30 years), were diagnosed from the general population through physical examination, and had a limited contribution to HIV transmission at the time of HIV diagnosis. These findings suggested that early detection, diagnosis, and intervention in patients with LHD may prevent further transmission of HIV.

## Data Availability

All data generated or analyzed during this study are included in this published article.
